# Blood purification could tackle COVID-19?

**DOI:** 10.1186/s40560-021-00586-0

**Published:** 2021-12-11

**Authors:** Hiroyuki Yamada, Shigeru Ohtsuru

**Affiliations:** 1 Department of Primary Care and Emergency Medicine, Graduate School of Medicine, Kyoto University, 54 Shogoin-kawaharacho, Sakyo-ku, Kyoto, 6068507 Japan; 2 Department of Nephrology, Graduate School of Medicine, Kyoto University, Kyoto, Japan

**Keywords:** Plasmapheresis, Plasma exchange, Cytosorb, Apheresis, AN69ST, Oxiris, PMX-DHP, Coronavirus, Pandemic, Cytokine

## Abstract

Coronavirus disease 2019 (COVID-19) threatened human lives worldwide since first reported. The current challenge for global intensivists is to establish an effective treatment for severe COVID-19. Blood purification has been applied to the treatment of various critical illnesses. Theoretically, its technique also has an enormous possibility of treating severe COVID-19 in managing inflammatory cytokines and coagulopathy. Recent clinical studies have revealed the positive clinical effect of therapeutic plasma exchange. Other studies have also indicated the considerable potential of other blood purification techniques, such as Cytosorb, AN69 surface-treated membrane, and polymyxin b hemoperfusion. Further research is needed to elucidate the actual effects of these applications.

## Background

Coronavirus disease 2019 (COVID-19) has been a new threat to humans since it was first reported in Wuhan, China. Despite the development of vaccines and medical treatment, a portion of COVID-19 patients become critically ill due to acute respiratory distress syndrome (ARDS) and other complications [1–4]. As recent basic studies indicate, these multiorgan dysfunctions mainly derive from three pathogeneses: cytokine storm, excessive inflammatory response, and hypercoagulation [5, 6]. To tackle this global crisis, we need to establish a treatment to control these pathological mechanisms.

Blood purification is a type of therapy based on the extracorporeal treatment of blood [7]. It has been widely used to treat severe refractory disorders to conventional therapies, such as fulminant liver failure, collagen diseases, and transplant rejection [8]. These applications have theoretical backgrounds such as suppressing excessive inflammation, lessening the cytokine storm, and correcting the coagulopathy. Many physicians are now wondering if this theoretical application could also be favorable in the treatment of patients with severe COVID-19. Based on this background, we focus on the positive possibility of blood purification in COVID-19.

## Main text

### Therapeutic plasma exchange

Therapeutic plasma exchange (TPE) is a blood purification therapy that efficiently separates the plasma from the blood cells and replaces it with fresh frozen plasma. Theoretically, the technique could both remove the pathological plasma with multiple cytokines and normalize the coagulopathy. Since the beginning of the COVID-19 pandemic, there have been case reports of the successful TPE treatment of patients with severe COVID-19 [9–12].These reports also demonstrated improved cytokine concentrations and coagulation markers immediately after the TPE procedure [10–12]. Clinical studies were also initiated to examine the clinical effect of TPE on severe COVID-19 cases (Table 1).

**Table 1:** Summary of the published studies investigating the effect of therapeutic plasma exchange for COVID-19

Trials	Study design	Sample size (TPE/control)	TPE replacement fluids	Number of TPE treatment	Results (mortality rate, MV, LOS)
Gucyetmez et al. [13]	Retrospective study with PSM	12/12	Not mentioned	Not mentioned	・ Mortality rate—mortality, 8% vs. 58%, p < 0.01 ・ MV—duration of MV, 316 h vs. 278 h, p = 0.67 ・LOS—ICU LOS, 20 days vs. 14 days, p = 0.07
Kamran et al. [14]	Retrospective study with PSM	45/45	FFP: normal saline = 2:1	Daily until recovery	・Mortality rate—overall survival, 91% vs. 62%, p < 0.01 ・ LOS—Total LOS, 10 days vs. 15 days, p = 0.01
Khamis et al. [15]	Retrospective study	11/20	FFP	5 times	・ Mortality rate—all-cause mortality, 9% vs. 45%, p = 0.055 28 day mortality, 0% vs. 35%, p = 0.033 14 day mortality, 0% vs. 35%, p = 0.033 ・ MV—extubation, 73% vs. 20%, p = 0.02 ・ LOS—total LOS, 19 days vs. 11 days, p = 0.13 ICU LOS, 14 days vs. 6 days, p = 0.03
Faqihi et al. [16]	RCT	34/39	FFP or artificial Ocyaplas LG®	Maximum of 5 times	・ Mortality rate—35 day mortality, 21% vs. 34%, p = 0.09 ・ MV—duration of MV, 15 days vs. 19 days, p < 0.01 ・ LOS—ICU LOS, 19 days vs. 26 days, p = 0.02

*APACHE* Acute Physiology and Chronic Health Evaluation, *ARDS* acute respiratory distress syndrome, *FFP* fresh frozen plasma, *ICU* intensive care unit, *LOS* length of stay, *MDOS* multiple organ dysfunction syndrome, *MV* mechanical ventilation, *TPE* therapeutic plasma exchange, *PSM* propensity score matching, *RCT* randomized controlled trial

Using a retrospective propensity score-matched analysis, Gucyetmez et al. compared the prognosis of COVID-19 patients in the intensive care unit (ICU) between patients receiving standard therapy alone and patients receiving standard therapy plus TPE [13]. As in previous reports, this study showed that TPE decreased interleukin-6 (IL-6) and D-dimer levels. Additionally, among patients with higher D-dimer (≧2) levels, the TPE group had a significantly lower mortality rate (8% vs. 58%; *p* < 0.01). Kamran et al. also applied a similar analysis to 280 COVID-19 patients and investigated the clinical effect of TPE [14]. They found that the TPE group’s 28-day survival rate was significantly superior (91% vs. 62%; *p* < 0.01). The median duration of hospitalization was also reduced in the TPE group (10 days vs. 15 days; *p* < 0.01). These retrospective studies suggested that TPE could improve the laboratory markers and ameliorate the prognosis of severe COVID-19 cases [13–15].

To address the same clinical question, Faqihi et al. designed a randomized controlled trial (RCT) that compared standard therapy alone with standard therapy plus TPE [16]. Although the 35-day mortality rate was not significantly lower in the TPE group (21% vs. 34%, *p* = 0.09), the duration of mechanical ventilation (15 days vs. 19 days; *p* < 0.01) and ICU stay (19 days vs. 26 days; *p* = 0.02) was significantly reduced. This RCT’s results also supported the efficacy of TPE for severe COVID-19 cases.

### Cytosorb

Cytosorb is a hemadsorption device that was approved in the European Union in 2011 for cytokine adsorption [17]. Its therapeutic impact on cytokine removal has been reported in various critical diseases, such as septic shock and cardiac surgery [18, 19]. One feature of this device is that it permits combination with other extracorporeal blood treatments, including continuous kidney replacement therapy (CKRT) and extracorporeal membrane oxygenation (ECMO). The co-treatment efficacy of Cytosorb and venous–venous ECMO (V–V ECMO) for severe COVID-19 pneumonia has been investigated in several clinical studies [20–22].

Rieder et al. preliminarily revealed that the initiation of V–V ECMO with Cytosorb markedly decreased the IL-6 level of COVID-19 patients [23]. They performed an open-label, multicenter RCT to evaluate the effect of V–V ECMO and Cytosorb for severe COVID-19-related ARDS [24]. In contrast to their preliminary data, however, their RCT’s results countered the authors’ expectations [25]. Namely, the 30-day survival rate was considerably lower in the Cytosorb group (18% vs. 76%; *p* < 0.01). Even the serum IL-6 level after 72 h was not significantly reduced (99 pg/mL vs. 112 pg/mL; *p* = 0.54). As such, they concluded that early Cytosorb initiation should be avoided in severe COVID-19 patients requiring V–V ECMO.

It is of note, however, that this RCT’s results have been questioned in regard to randomization, timing of ECMO, and serum IL-6 concentrations [26, 27]. Therefore, it might be premature to rush to negative conclusions about the efficacy of Cytosorb. There are other RCTs that are currently examining the effects, such as NCT04518969, NCT04344080, DRKS00021447, and further investigations are expected [28].

### Modified AN69ST (Oxiris) and polymyxin b hemoperfusion

Oxiris is a newly developed CKRT hemofilter with an AN69 surface-treated membrane [29]. It provides high absorbance of endotoxin (negatively charged) and excellent anti-thrombogenicity because of its positively charged polyethyleneimine coating and heparin grafting [30]. Several case series and studies have already reported the hemofilter’s validity in reducing cytokine concentrations in COVID-19 patients [31–35].

Polymyxin b hemoperfusion (PMX-DHP) is another widely used blood purification therapy for septic shock patients [36]. The hemoperfusion therapy removes circulating endotoxins through the adsorption to polymyxin b-immobilized columns. Although the treatment efficacy was not supported by recent guidelines, some case series have shown the clinical value of PMX-DHP for COVID-19 patients [37–40]. Other studies also revealed that PMX-DHP could decrease levels of IL-6 and other inflammatory chemokines [41].

Unfortunately, the efficacy of these two therapies has not yet been sufficiently examined with control studies or RCTs. In vitro examinations indicate that these techniques could calm cytokine storms, however, and future research is thus warranted.

## Conclusions

Blood purification has excellent potential to fight the COVID-19 pandemic. TPE, in particular, could be helpful in the clinical management of cytokine storms and coagulopathy. The efficacy of other techniques has also been supported by several clinical and in vitro studies. Further research is needed to elucidate the actual effects of these applications.

## Data Availability

All descriptions are based on the published data.

## Abbreviations

ARDS: Acute respiratory distress syndrome
CKRT: Continuous kidney replacement therapy
COVID-19: Coronavirus disease 2019
ECMO: Extracorporeal membrane oxygenation
ICU: Intensive care unit
PMX-DHP: Polymyxin b hemoperfusion
RCT: Randomized controlled trial
TPE: Therapeutic plasma exchange
